# PLCβ-Mediated Depletion of PIP_2_ and ATP-Sensitive K^+^ Channels Are Involved in Arginine Vasopressin-Induced Facilitation of Neuronal Excitability and LTP in the Dentate Gyrus

**DOI:** 10.1523/ENEURO.0120-22.2022

**Published:** 2022-07-18

**Authors:** Saobo Lei, Cody A. Boyle, Morgan Mastrud

**Affiliations:** Department of Biomedical Sciences, School of Medicine and Health Sciences, University of North Dakota, Grand Forks, North Dakota 58203

**Keywords:** action potential, depolarization, hippocampus, peptide, receptors, signal transduction

## Abstract

Arginine vasopressin (AVP) serves as a neuromodulator in the brain. The hippocampus is one of the major targets for AVP, as it has been demonstrated that the hippocampus receives vasopressinergic innervation and expresses AVP receptors. The dentate gyrus (DG) granule cells (GCs) serve as a gate governing the inflow of information to the hippocampus. High densities of AVP receptors are expressed in the DG GCs. However, the roles and the underlying cellular and molecular mechanisms of AVP in the DG GCs have not been determined. We addressed this question by recording from the DG GCs in rat hippocampal slices. Our results showed that application of AVP concentration-dependently evoked an inward holding current recorded from the DG GCs. AVP depolarized the DG GCs and increased their action potential firing frequency. The excitatory effects of AVP were mediated by activation of V_1a_ receptors and required the function of phospholipase Cβ (PLCβ). Whereas intracellular Ca^2+^ release and protein kinase C activity were unnecessary, PLCβ-induced depletion of phosphatidylinositol 4,5-bisphosphate was involved in AVP-evoked excitation of the DG GCs. AVP excited the DG GCs by depression of the ATP-sensitive K^+^ channels, which were required for AVP-elicited facilitation of long-term potentiation at the perforant path–GC synapses. Our results may provide a cellular and molecular mechanism to explain the physiological functions of AVP, such as learning and memory, and pathologic disorders like anxiety.

## Significance Statement

Dentate gyrus is the first station of the hippocampus and serves as the gate governing the inflow of information to the hippocampus. Modification of the excitability of the dentate gyrus granule cells likely plays a significant role in the expression of hippocampal functions. We showed that activation of V_1a_ receptors excites dentate gyrus granule cells by phospholipase Cβ-mediated depression of ATP-sensitive K^+^ channels and that this cellular mechanism is responsible for arginine vasopressin (AVP)-elicited facilitation of long-term potentiation. Our results may provide a cellular and molecular mechanism to explain the physiological functions of AVP, such as learning and memory, and pathologic disorders like anxiety.

## Introduction

Arginine vasopressin (AVP) is a nonapeptide synthesized in the paraventricular and supraoptic nuclei of the hypothalamus. AVP is then transported along the axons of these neurosecretory cells to the posterior pituitary where it is released into the bloodstream to exert its hormonal functions in the periphery on blood vessels, kidney, and uterus (Stoop, 2012). Additionally, vasopressinergic fibers from the parvocellular neurons of the hypothalamus project to discrete extrahypothalamic limbic brain regions including the hippocampus, subiculum, amygdala, and nucleus accumbens (Buijs et al., 1978; Buijs and Swaab, 1979; Lang et al., 1983; DeVries et al., 1985; Hawthorn et al., 1985). While the hypothalamus and pituitary form the major source of AVP in the brain, AVP immunoreactivity has also been detected in neurons in the extrahypothalamic structures, including the bed nucleus of stria terminalis, septal region, medial amygdala, and locus coeruleus (Caffé and van Leeuwen, 1983; van Leeuwen and Caffé, 1983; Sofroniew, 1985), although the targets of these vasopressinergic projections have not been clearly defined.

The biological functions of AVP are mediated by interacting with three types of vasopressin receptors: V_1a_, V_1b_, and V_2_ receptors. V_1a_ and V_1b_ receptors are coupled to the Gα_q/11_ proteins activating phospholipase Cβ (PLCβ), which further breaks down phosphatidylinositol 4,5-bisphosphate (PIP_2_) to generate 1,4,5-trisphosphate (IP_3_) to elevate intracellular Ca^2+^ release and diacylglycerol (DAG) to activate protein kinase C (PKC). V_2_ receptors are coupled to G_s_-proteins, increasing the activity of adenylyl cyclase to elevate cyclic AMP levels. In the brain, AVP serves as a neuromodulator that regulates a variety of physiological functions including social behaviors (Cilz et al., 2019; Kompier et al., 2019), learning and memory (de Wied et al., 1993; Caldwell et al., 2008), nociception (Koshimizu and Tsujimoto, 2009), circadian rhythms (Gizowski et al., 2017), and neurologic diseases such as anxiety (Caldwell et al., 2008; Neumann and Landgraf, 2012). However, the cellular and molecular mechanisms whereby AVP modulates these physiological functions and pathologic disorders have not been completely determined.

The hippocampus is one of the major biological targets for AVP because high densities of vasopressin receptors have been detected in the hippocampus (Biegon et al., 1984; Brinton et al., 1984; De Kloet et al., 1985; Lawrence et al., 1988) and the hippocampus also receives vasopressinergic innervation (Buijs, 1980; Caffé et al., 1987; Metzger et al., 1993). In line with the distributions of both AVP-containing fibers and AVP receptors in the hippocampus, activation of V_1a_ receptors excites both pyramidal neurons (Hu et al., 2022) and interneurons (Ramanathan et al., 2012) in the CA1 region. However, the highest densities of AVP receptors (Brinton et al., 1984; De Kloet et al., 1985; van Leeuwen et al., 1987; Campbell et al., 2009), especially the V_1a_ receptors (Ostrowski et al., 1994; Szot et al., 1994), have been detected in the dentate gyrus (DG), which serves as the gate governing the inflow of information to the hippocampus. Consistent with the anatomic expression of AVP receptors in the DG, bath application of AVP modulates the slope of the field potentials in the DG recorded from *in vitro* slices, depending on the extracellular Ca^2+^ concentration (Chen et al., 1993). Furthermore, intracerebroventricular injection of AVP augments long-term potentiation (LTP) in the DG in intact anesthetized rats (Dubrovsky et al., 2003) and induces Fos protein expression in the DG (Paban et al., 1999), suggesting that AVP increases the neuronal excitability in the DG. However, the cellular and molecular mechanisms whereby AVP modulates neuronal excitability and synaptic transmission and plasticity in the DG have not been determined. In this study, we studied the effects of AVP on the excitability of the granule cells (GCs) in the DG. Our results indicate that the activation of V_1a_ receptors increases the excitability of the DG GCs via PLCβ-mediated depression of the ATP-sensitive K^+^ (K_ATP_) channels. AVP did not modulate glutamatergic transmission but augmented the LTP at the perforant path (PP)–GC synapses. Our results may provide a cellular and molecular mechanism to explain the functions of AVP *in vivo*.

## Materials and Methods

### Slice preparation

Horizontal brain slices (350 μm) were prepared from both male and female Sprague Dawley rats (25–40 d old) purchased from Envigo RMS. Animals were housed in the institutional animal center with food and water available *ad libitum* until use. The animal rooms were maintained on a 14/10 h light/dark cycle (lights on at 7:00 A.M.), with a room temperature of 22°C. All procedures and experiments presented in this study were approved by the Institutional Animal Care and Use Committee and performed in accordance with the National Institutes of Health *Guide for the Care and Use of Laboratory Animals*. The number of males and females for each experiment was kept as equal as possible. After being deeply anesthetized with isoflurane, an animal was decapitated and the brain was dissected out. Slices were cut in ice-cold saline solution that contained the following (in mm): 250 glycerol, 2.5 KCl, 1.2 NaH_2_PO_4_, 1.2 MgCl_2_, 2.4 CaCl_2_, 26 NaHCO_3_, and 11 glucose, at ∼330 mOsm, as described previously (Ye et al., 2006). After incubation at 35°C for 60 min in the extracellular solution containing (in mm) 130 NaCl, 24 NaHCO_3_, 3.5 KCl, 1.25 NaH_2_PO_4_, 2.5 CaCl_2_, 1.5 MgCl_2_, and 10 glucose, saturated with 95% O_2_ and 5% CO_2_, slices were kept at room temperature until use. All animal procedures conformed to the guidelines approved by the Institutional Animal Care and Use Committee.

### Recordings of action potentials, resting membrane potentials, and holding currents from the DG GCs

Whole-cell recordings using a Multiclamp 700B amplifier (Molecular Devices) in voltage-clamp or current-clamp mode were made from the DG GCs visually identified with infrared video microscopy (model BX51WI microscope, Olympus) and differential interference contrast optics. During recordings, the bath temperature was maintained at 33–34°C by an inline heater and an automatic temperature controller (model TC-324C, Warner Instruments). The bath solution was the above-mentioned incubation extracellular solution. The recording electrodes were filled with the following (in mm): 120 K^+^-gluconate, 10 KCl, 2 MgCl_2_, 10 HEPES, 0.6 EGTA, 2 ATPNa_2_, 0.4 GTPNa, and 5 phosphocreatine, at pH 7.3, unless stated otherwise. Holding currents at −60 mV and resting membrane potentials (RMPs) were recorded in the extracellular solution supplemented with tetrodotoxin (TTX; 0.5 μm), kynurenic acid (1 mm), and picrotoxin (100 μm) to block action potential (AP) firing, glutamatergic transmission, and GABAergic transmission, respectively. APs evoked by injections of a series of positive currents from 25 to 400 pA at an interval of 25 pA were recorded in the above solution without TTX. AVP was dissolved in the extracellular solution and bath applied. To avoid potential desensitization induced by repeated applications of the agonist, one slice was limited to only one application of AVP. Pharmacological inhibitors were applied to the cells either extracellularly or intracellularly via the recording pipettes. For extracellular application, slices were pretreated for at least 1 h to ensure permeation of reagents into the cells in the slices and the extracellular solution continuously contained the same concentration of the reagents, unless stated otherwise. For intracellular application, we waited for >15 min after the formation of whole-cell configuration to ensure the diffusion of the inhibitors into the cells. Data were filtered at 2 kHz, digitized at 10 kHz, acquired, and analyzed subsequently using pCLAMP 10.7 software (Molecular Devices).

### Recordings of AMPA EPSCs and LTP

Whole-cell recordings were used to record AMPA EPSCs at −65 mV from the DG GCs by placing a concentric bipolar stimulation electrode [model MX21XES(DB9), FHC] in the middle to the inner one-third of the molecular layer of the DG to stimulate the medial PP. The intracellular solution was the above K^+^-gluconate internal solution supplemented with QX-314 (1 mm) to block AP firing. For a subset of experiments, a Cs^+^-containing intracellular solution was prepared by replacing the K^+^ in the above solution with the same concentration of Cs^+^ that was used. The extracellular solution was supplemented with 10 μm bicuculline to block GABAergic transmission. The stimulation intensity was set to the level that produced 30–40% of the maximal amplitude of EPSCs. After recording basal AMPA EPSCs at −65 mV in 0.1 Hz, we applied a protocol by paring presynaptic stimulation (1 Hz, for 40 pulses) with postsynaptic depolarization to −30 mV to induce LTP, as described previously (Colino and Malenka, 1993). Recordings of AMPA EPSCs (at −65 mV in 0.1 Hz) were resumed after the protocol to monitor LTP. The amplitudes of AMPA EPSCs were normalized to the average of those recorded in control condition for 5 min. Series resistance was rigorously monitored by the delivery of 5 mV voltage steps after each evoked current. Experiments were discontinued if the series resistance changed by >15%.

### Data analysis

Data were presented as the mean ± SEM. The concentration–response curve of AVP was fit by the Hill equation: *I* = *I*_max_ × {1/[1 + (EC_50_/[ligand])*^n^*]}, where *I*_max_ is the maximum response, EC_50_ is the concentration of ligand producing a half-maximal response, and *n* is the Hill coefficient. *N* numbers in the text were the numbers of cells used for each experiment. To minimize potential influences of variation from individual animals, at least four animals were used for each experiment. Because the maximal response occurred within 5 min during the application of AVP, we measured the peak response of AVP for statistical analysis. A Wilcoxon matched-pairs signed-rank test (abbreviated as “Wilcoxon test” in the text), Mann–Whitney test, one-way ANOVA followed by Dunnett’s multiple-comparisons test, or two-way repeated-measures ANOVA followed by a Sidak multiple-comparisons test was used as appropriate for statistical analysis. The control data for AVP-induced inward currents were pooled results from the control experiments performed for each individual pharmacological experiment. A one-way ANOVA followed by Dunnett’s multiple-comparisons test was used for statistical analysis when the pooled control data were used for comparison. The *p* values were reported throughout the text, and significance was set as *p *<* *0.05.

### Chemicals

The following chemicals were products of R&D Systems: AVP, TTX, kynurenic acid, picrotoxin, SR49059, U73122, heparin, thapsigargin, bisindolylmaleimide II (Bis II), RHC 80267, and ML 133. Glibenclamide was purchased from MedChemExpress. diC8-PIP_2_ was purchased from Echelon Biosciences. Drugs were initially prepared in stock solution, aliquoted, and stored at −20°C. For those chemicals requiring dimethylsulfoxide (DMSO) as a solvent, the concentration of DMSO was <0.1%.

## Results

### AVP elicits an inward current in the DG GCs via activation of V_1a_ receptors

**Figure 1.**
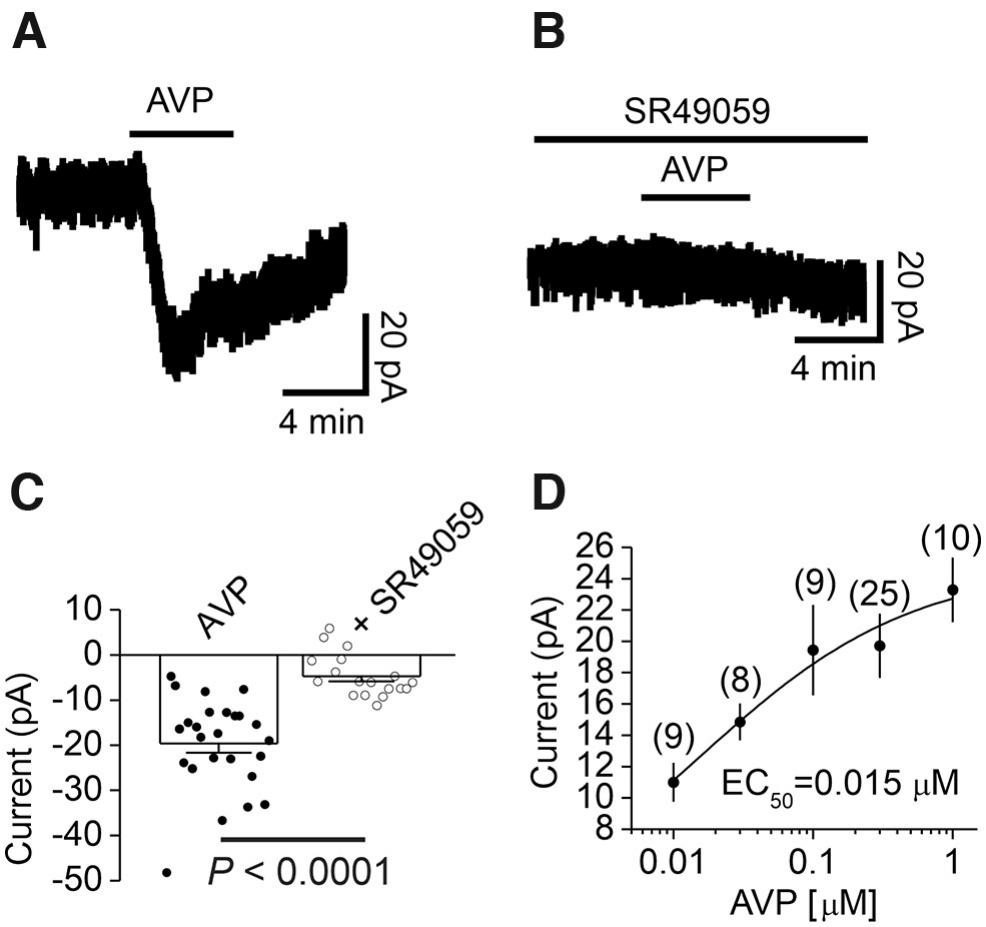
Bath application of AVP elicits an inward current in DG GCs. ***A***, Current trace recorded from a DG GC in response to bath application of AVP (0.3 μm). ***B***, Current trace recorded from a DG GC in a slice pretreated with the selective V_1a_ receptor antagonist SR49095 (1 μm). The extracellular solution continuously contained the same concentration of SR49095. ***C***, Summary graph showing AVP-induced inward currents in control condition or in the presence of SR49095 (1 μm). The circles represent the values from individual cells, and the bars are their averages. ***D***, Concentration–response curve of AVP constructed by measuring AVP-induced inward currents. The numbers within the parentheses were the numbers of cells recorded at each concentration.

We probed the effects of AVP on the DG GCs by recording the holding currents at −60 mV in voltage clamp. Bath application of AVP (0.3 μm) evoked an inward current (−19.7 ± 2.1 pA, *n* = 25; *p *<* *0.0001 vs baseline, Wilcoxon test; Fig. 1*A*,*C*). The effect of AVP was mediated by activation of V_1a_ receptors because pretreatment of slices with and continuous bath application of the selective V_1a_ receptor antagonist SR49059 (1 μm) significantly reduced AVP-evoked inward currents (−4.6 ± 1.1 pA, *n* = 18; *p *=* *0.002 vs baseline, Wilcoxon test; *p *<* *0.0001 vs AVP alone, Mann–Whitney test; Fig. 1*B*,*C*), suggesting the involvement of V_1a_ receptors. The EC_50_ of AVP was calculated to be 0.015 μm (Fig. 1*D*). We used AVP at 0.3 μm for the remaining experiments because this is a near-saturating concentration.

### AVP-elicited inward currents in the DG GCs are mediated by depressing K_ATP_ channels

**Figure 2.**
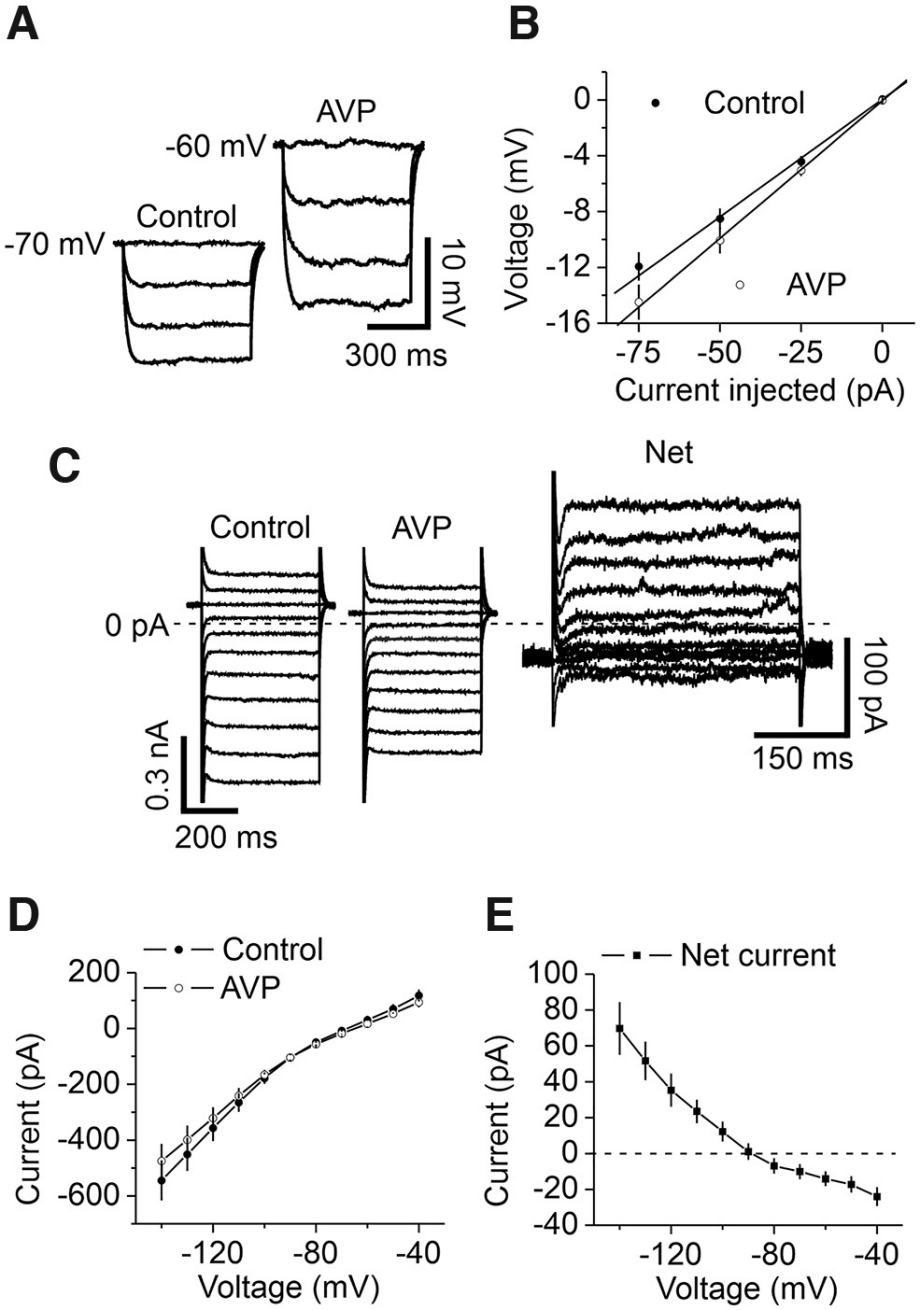
AVP-elicited inward currents are mediated by depression of Kir channels. ***A***, ***B***, AVP increased the input resistance of DG GCs. ***A***, Voltage responses evoked by the injection of negative currents from 0 to −75 pA at an interval of 25 pA before (left) and during (right) the application of AVP from a DG GC. ***B***, The current–voltage relationship averaged from nine cells. Input resistance was obtained by linear fitting of the current–voltage relationship. ***C***, Currents elicited by a voltage step protocol before (left) and during (middle) bath application of AVP and the net current obtained by subtraction (right) from a GC. Cells were held at −60 mV and stepped from −140 to −40 mV for 400 ms at a voltage interval of 10 mV every 10 s. Steady-state currents were measured within 5 ms before the end of the step voltage protocols. Note the differences in the scale bars. The dashed line was the zero current level. ***D***, *I–V* curve averaged from 12 GCs before and during the application of AVP. ***E***, *I–V* curve of the net current obtained by subtracting the currents in control condition from those during the application of AVP. Note that the net currents showed inward rectification, suggesting the involvement of Kir channels.

We further determined the ionic mechanisms underlying AVP-elicited inward currents in the DG GCs. We measured the input resistance (R_in_) of the DG GCs before and during the application of AVP by injecting negative currents from 0 to −75 pA with 25 pA steps for a duration of 600 ms. We fit the current–voltage relationship (*I–V*) with a linear function for each cell to obtain R_in_, which equals the slope of the linear fitting. Bath application of AVP depolarizes DG GCs (see below) and increased R_in_ (control, 155 ± 15 MΩ; AVP, 188 ± 18 MΩ; *n* = 9; *p *=* *0.004, Wilcoxon test; Fig. 2*A*,*B*), suggesting that AVP decreases a membrane conductance. We further measured the *I–V* of the currents generated by AVP. The extracellular solution was supplemented with TTX (0.5 μm) to block voltage‐gated Na^+^ channels. Cells were held at −60 mV and stepped from −140 to −40 mV for 400 ms at a voltage interval of 10 mV every 10 s. Steady‐state currents were measured within 5 ms before the end of the step voltage protocol. Under these circumstances, the *I–V* curve of the AVP‐elicited currents recorded from the DG GCs showed inward rectification with a reversal potential of −90.4 ± 3.4 mV (*n* = 12; Fig. 2*C–E*), resembling that of the inwardly rectifying K^+^ (Kir) channels. This result suggests that AVP-induced inward currents are mediated by inhibiting a Kir channel.

**Figure 3.**
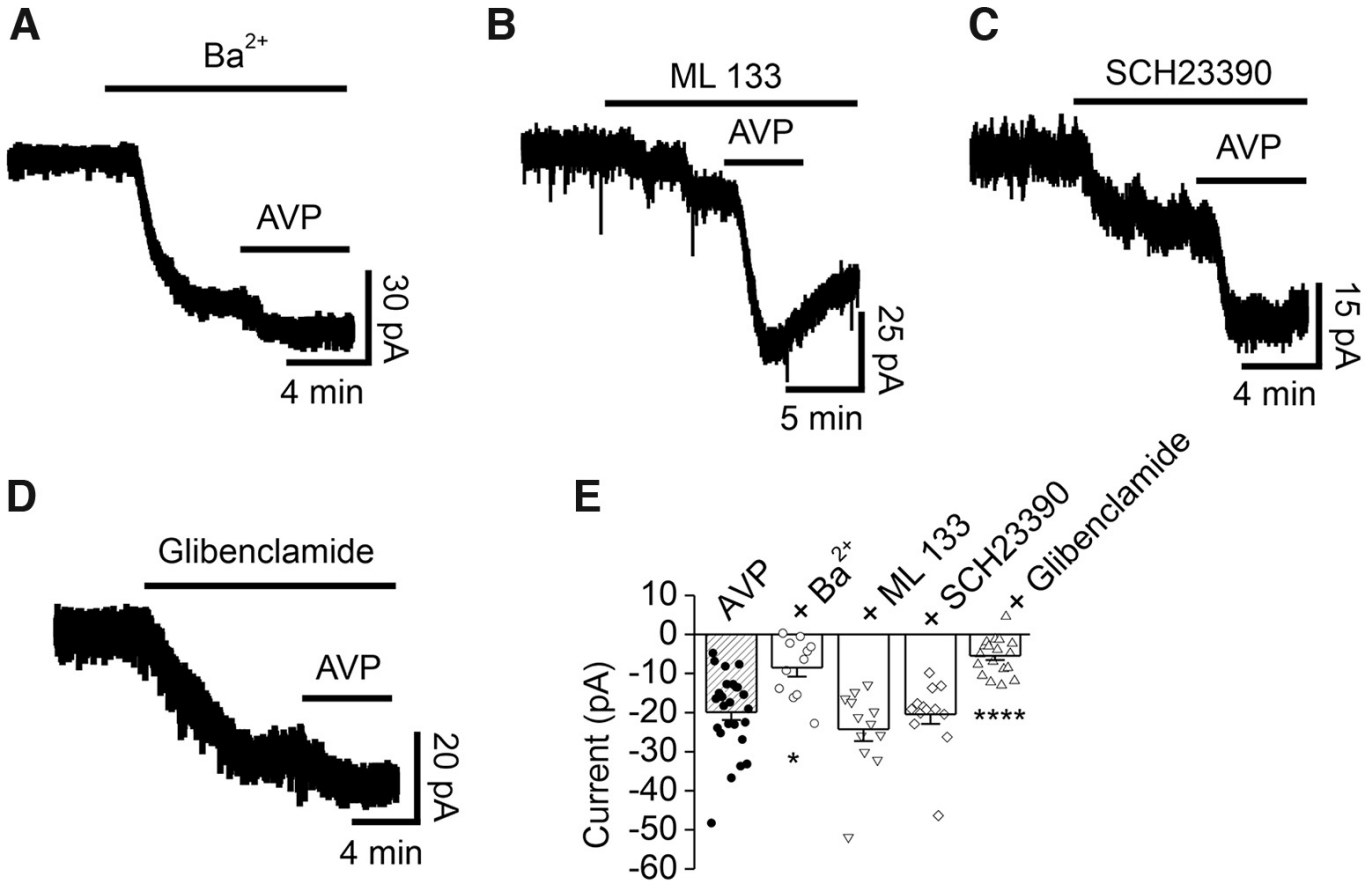
Effects of Kir channel blockers on AVP-elicited inward currents recorded from DG GCs. ***A***, Current trace recorded from a DG GC in response to bath application of Ba^2+^ (500 μm) alone and concomitant application of AVP. ***B***, Current trace recorded from a DG GC in response to bath application of ML 133 (30 μm) alone and coapplication of AVP. ***C***, Current trace recorded from a DG GC in response to bath application of SCH23390 (40 μm) alone and coapplication of AVP. ***D***, Current trace recorded from a DG GC in response to bath application of glibenclamide (100 μm) alone and coapplication of AVP. ***E***, Summary graph showing the effects of Kir channel blockers on AVP-induced inward currents. The shaded bar was the averaged inward currents evoked by AVP in control condition pooled from the control experiment conducted for each individual pharmacological experiment. *p =* *0.018, ****p <* *0.0001 versus AVP alone, one-way ANOVA followed by Dunnett’s test.

We further confirmed the involvement of Kir channels in AVP-induced inward currents in the DG GCs with Kir channel blockers. Bath application of Ba^2+^ (500 μm) induced an inward current by itself (−55.0 ± 10.7 pA, *n* = 11; *p *=* *0.001 vs baseline, Wilcoxon test; Fig. 3*A*), suggesting that the DG GCs express functional Kir channels. Following application of AVP in the presence of Ba^2+^ evoked a significantly smaller inward current (−8.5 ± 2.3 pA, *n* = 11; *p *=* *0.002 vs Ba^2+^ alone, Wilcoxon test; *p *=* *0.018 vs AVP alone, one-way ANOVA followed by Dunnett’s test; Fig. 3*A*,*E*), further confirming the involvement of Kir channels. Kir channels are classified into four functional groups including Kir2, Kir3 [G-protein-gated Kir (GIRK) channels], Kir6 (K_ATP_ channels) and K^+^ transport channels (Hibino et al., 2010). We used ML 133, a specific antagonist for Kir2 subfamily channels (Wang et al., 2011; Kim et al., 2015; Ford and Baccei, 2016; Sonkusare et al., 2016; Huang et al., 2018), to test the roles of the Kir2 subfamily channels in AVP-elicited inward currents. Bath application of ML 133 (30 μm) by itself evoked a small inward current (−6.7 ± 1.3 pA, *n* = 12; *p *=* *0.001 vs baseline, Wilcoxon test; Fig. 3*B*). Subsequent application of AVP still elicited a comparable inward current (−24.3 ± 3.0 pA, *n* = 12; *p *=* *0.0005 vs ML 133 alone, Wilcoxon test; *p *=* *0.808 vs AVP alone, one-way ANOVA followed by Dunnett’s test; Fig. 3*B*,*E*), suggesting that the Kir2 subfamily is not involved in AVP-induced inward currents.

We further tested the roles of the Kir3 subfamily in AVP-elicited inward currents in the DG GCs. Bath application of the Kir3 channel blocker SCH23390 (40 μm; Kuzhikandathil and Oxford, 2002) evoked an inward current by itself (−29.7 ± 5.1 pA, *n* = 13; *p *=* *0.0002 vs baseline, Wilcoxon test; Fig. 3*C*), suggesting the expression of Kir3 channels in the DG GCs. However, application of AVP in the presence of SCH23390 still elicited a comparable inward current (−20.5 ± 2.5 pA, *n* = 13; *p *=* *0.0002 vs SCH23390 alone, Wilcoxon test; *p *=* *0.999 vs AVP alone, one-way ANOVA followed by Dunnett’s test; Fig. 3*C*,*E*), suggesting that the Kir3 subfamily is not involved in AVP-induced inward currents.

Whereas our intracellular solution in the recording pipettes contained 2 mm ATP, which should exert inhibition on K_ATP_ channels, the effects of K_ATP_ channels on neuronal excitability are not fully blocked by an intracellular solution containing 4 mm ATP (Lemak et al., 2014), possibly because the open probability of K_ATP_ channels reflects activity-dependent fluctuations of ATP/ADP concentrations within local submembrane domains that are not entirely controlled by the solution in the patch pipette (Haller et al., 2001; Mollajew et al., 2013). Furthermore, as will be shown below, PLCβ-mediated depletion of PIP_2_ was involved in AVP-mediated inward currents, and PIP_2_ alters the sensitivity of K_ATP_ channels to ATP (Hilgemann and Ball, 1996; Fan and Makielski, 1997; Baukrowitz et al., 1998; Shyng and Nichols, 1998). Another rationale to test the roles of K_ATP_ channels is that high densities of K_ATP_ channels are expressed in the DG GCs (Mourre et al., 1990; Zawar et al., 1999; Pelletier et al., 2000; Tanner et al., 2011). We thus probed the roles of K_ATP_ channels in AVP-induced inward currents by testing the hypothesis that activation of V_1a_ receptors generates an inward current by depressing K_ATP_ channels in the DG GCs. The premise of this hypothesis is that K_ATP_ channels should be open in the resting condition. We therefore used the selective K_ATP_ channel blocker glibenclamide. Bath application of glibenclamide (100 μm) by itself induced an inward current (−22.8 ± 3.8 pA, *n* = 18; *p *<* *0.0001 vs baseline, Wilcoxon test; Fig. 3*D*), suggesting a tonic activation of K_ATP_ channels in the DG GCs. Following application of AVP in the continuous presence of glibenclamide generated a significantly smaller inward current (−5.6 ± 1.1 pA, *n* = 18; *p *=* *0.0002 vs glibenclamide alone, Wilcoxon test; *p *<* *0.0001 vs AVP alone, one-way ANOVA followed by Dunnett’s test; Fig. 3*D*,*E*), suggesting that AVP-generated inward currents were mediated by depressing K_ATP_ channels in the DG GCs.

### PLCβ, but not PKC or intracellular Ca^2+^ release is necessary for AVP-elicited inward currents in the DG GCs

**Figure 4.**
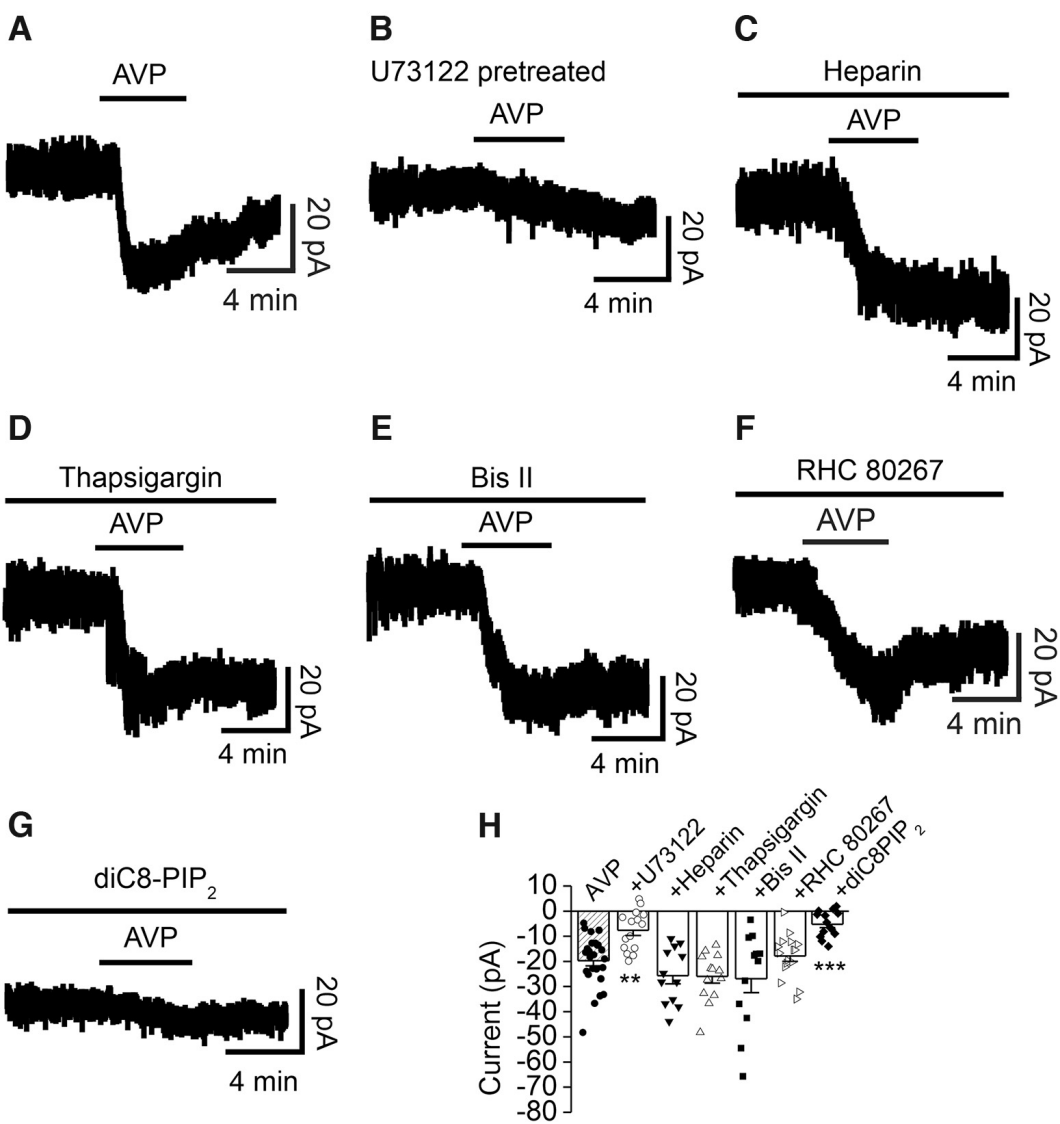
AVP-elicited inward currents depend on PLCβ and depletion of PIP_2_, but do not require the functions of intracellular Ca^2+^ release and PKC activity. ***A***, Current trace recorded from a DG GC in response to bath application of AVP alone. ***B***, Current trace recorded from a DG GC in response to bath application of AVP in a slice pretreated with the PLC inhibitor U73122 (5 μm). ***C***, Current trace recorded from a DG GC before, during, and after bath application of AVP in the intracellular solution containing heparin (0.5 mg/ml). ***D***, Current trace recorded from a DG GC in response to bath application of AVP in the intracellular solution supplemented with thapsigargin (10 μm). ***E***, AVP-induced inward current recorded from a DG GC in a slice pretreated with Bis II (2 μm). The extracellular solution continuously contained the same concentration of Bis II. ***F***, AVP-elicited inward current trace recorded from a DG GC in a slice pretreated with RHC 80267 (25 μm), a DAG lipase inhibitor. The extracellular solution contained the same concentration of RHC 80267. ***G***, Current trace recorded from a DG GC dialyzed with the intracellular solution containing diC8-PIP_2_ (50 μm). ***H***, Summary graph. The shaded bar was the averaged inward currents evoked by AVP in control condition pooled from the control experiment conducted for each individual pharmacological experiment. **p =* *0.002, ***p =* *0.0002 versus AVP alone, one-way ANOVA followed by Dunnett’s test.

V_1a_ receptors are coupled to Gα_q/11_ proteins elevating the activity of PLCβ, which hydrolyzes PIP_2_ to generate IP_3_ to increase intracellular Ca^2+^ release and DAG to activate PKC. We tested the roles of these signaling molecules in AVP-mediated inward currents in the DG GCs. Pretreatment of slices with and continuous bath application of the PLC inhibitor U73122 (5 μm) significantly reduced AVP-induced inward currents (−7.6 ± 2.0 pA, *n* = 15; *p *=* *0.003 vs baseline, Wilcoxon test; *p *=* *0.002 vs AVP alone, one-way ANOVA followed by Dunnett’s test; Fig. 4*B*,*H*), suggesting the involvement of PLCβ. We then tested the roles of intracellular Ca^2+^ released from the IP_3_ store and PKC in AVP-elicited inward currents. Dialysis of the IP_3_ receptor blocker heparin at an effective concentration (0.5 mg/ml; Saleem et al., 2014), via the recording pipettes, failed to alter significantly AVP-induced inward currents (−25.6 ± 3.2 pA, *n* = 12; *p *=* *0.0005 vs baseline, Wilcoxon test; *p *=* *0.523 vs AVP alone, one-way ANOVA followed by Dunnett’s test; Fig. 4*C*,*H*), suggesting that Ca^2+^ released from the IP_3_ store is not required for AVP-induced inward currents. Likewise, intracellular application of the endoplasmic reticulum Ca^2+^-ATPase inhibitor thapsigargin (10 μm) via the recording pipettes did not significantly change AVP-induced inward currents (−26.1 ± 2.7 pA, *n* = 13; *p = *0.0002 vs baseline, Wilcoxon test; *p *=* *0.378 vs AVP alone, one-way ANOVA followed by Dunnett’s test; Fig. 4*D*,*H*), suggesting that intracellular Ca^2+^ release is unnecessary for AVP-mediated inward currents. We further examined the roles of PKC in AVP-induced inward currents. Pretreatment of slices with and continuous bath application of the selective PKC inhibitor Bis II (1 μm) failed to block AVP‐elicited inward currents (−26.9 ± 5.5 pA, *n* = 12; *p *=* *0.0005 vs baseline, Wilcoxon test; *p *=* *0.282 vs AVP alone, one-way ANOVA followed by Dunnett’s test; Fig. 4*E*,*H*), suggesting that the function of PKC is not involved in AVP-induced inward currents.

DAG generated in response to the activation of G_q_-coupled receptors can be metabolized by DAG lipase to produce 2-arachidonoylglycerol (2-AG), which has been reported to inhibit A-type K^+^ channels to excite midbrain dopamine neurons (Gantz and Bean, 2017). We next tested the role of DAG lipase in AVP-elicited excitation of DG GCs. To exclude contributions from differing isoforms of DAG lipase, we used RHC 80267 to inhibit both α and β DAG lipase. Pretreatment of slices with and continuous bath application of RHC 80267 (25 μm) had no significant effect on AVP-induced inward currents (−17.9 ± 2.2 pA, *n* = 16; *p *<* *0.0001 vs baseline, Wilcoxon test; *p *=* *0.999 vs AVP alone, one-way ANOVA followed by Dunnett’s test; Fig. 4*F*,*H*), suggesting that AVP-elicited excitation of DG GCs is not mediated by 2-AG.

### Depletion of PIP_2_ is required for AVP-evoked inward currents in the DG GCs

PIP_2_ has been shown to modulate numerous ion channels (Suh and Hille, 2008; Rodríguez-Menchaca et al., 2012), including the K_ATP_ channels (Hilgemann and Ball, 1996; Fan and Makielski, 1997; Baukrowitz et al., 1998; Shyng and Nichols, 1998). We therefore studied the roles of PIP_2_ depletion elicited by activation of PLCβ in response to V_1a_ receptor activation. Inclusion of the short-chain, water-soluble analog diC8-PIP_2_ (50 μm) in the recording pipettes significantly reduced AVP-induced inward currents (−5.2 ± 1.4 pA, *n* = 14; *p *=* *0.004 vs baseline, Wilcoxon test; *p *=* *0.0002 vs AVP alone, one-way ANOVA followed by Dunnett’s test; Fig. 4*G*,*H*), suggesting that depletion of PIP_2_ is required for AVP-mediated inward currents in the DG GCs.

### Activation of V_1a_ receptors augments the excitability of the DG GCs

**Figure 5.**
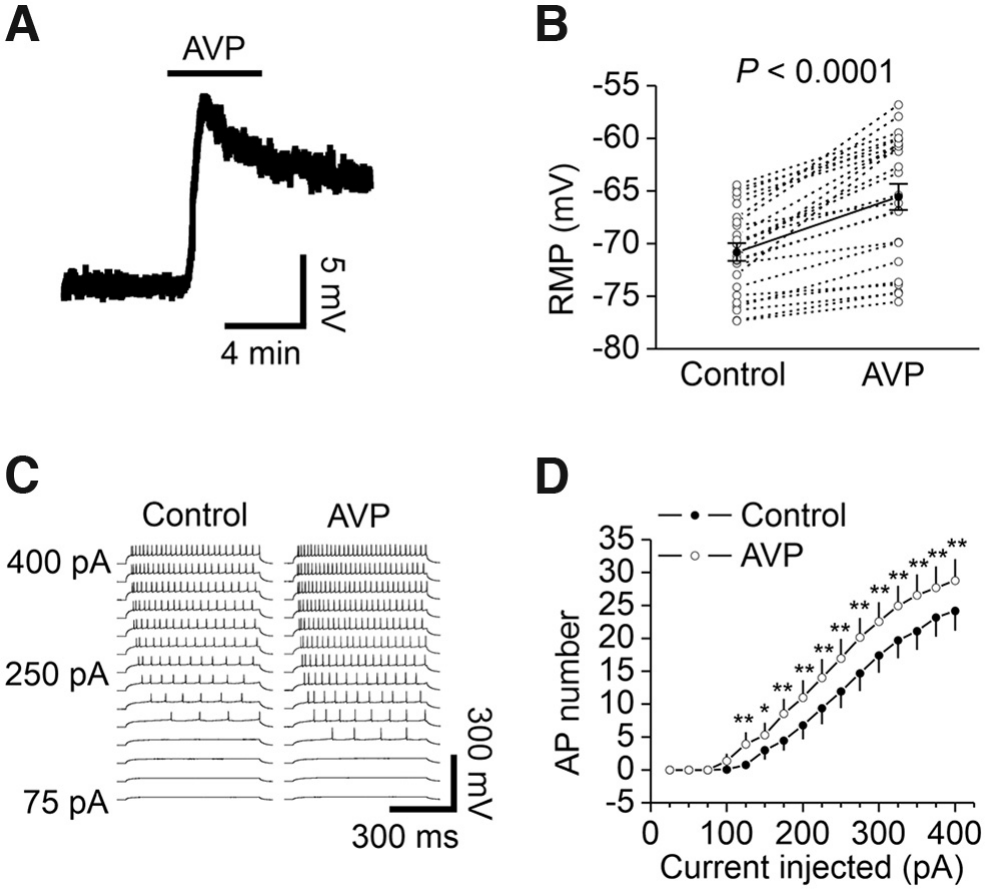
AVP depolarizes GCs and increases the number of APs elicited by injection of a series of positive currents. ***A***, Resting membrane potential recorded from a GC before, during, and after the application of AVP. ***B***, Summary data for AVP-induced depolarization. The empty circles represented the values from individual cells, and the solid symbols were their averages. ***C***, APs elicited by injections of a series of positive currents from 25 to 400 pA in a GC before (left) and during (right) the application of AVP. ***D***, Relationship between the injected currents and the elicited AP numbers from 13 GCs. *p <* *0.05, **p <* *0.001, two-way repeated-measures ANOVA followed by Sidak’s multiple-comparisons test.

We tested the effects of AVP on the RMPs and AP firing numbers recorded from the DG GCs. Bath application of AVP induced significant depolarization of the DG GCs (control, −70.8 ± 4.1 mV; AVP, −65.6 ± 6.0 mV; net depolarization, 5.2 ± 3.2 mV; *n* = 24; *p *<* *0.0001, Wilcoxon test; Fig. 5*A*,*B*). We further probed the effects of AVP on the excitability of GCs by measuring the number of APs evoked by injecting a series of positive currents from 25 to 400 pA at an interval of 25 pA. With this protocol, the application of AVP significantly enhanced the AP firing numbers (*F*_(1,12)_ = 24.05, *p *<* *0.001, two-way repeated-measures ANOVA followed by Sidak’s multiple-comparisons test; Fig. 5*C*,*D*).

### AVP does not modulate glutamatergic transmission at the PP–GC synapses

**Figure 6.**
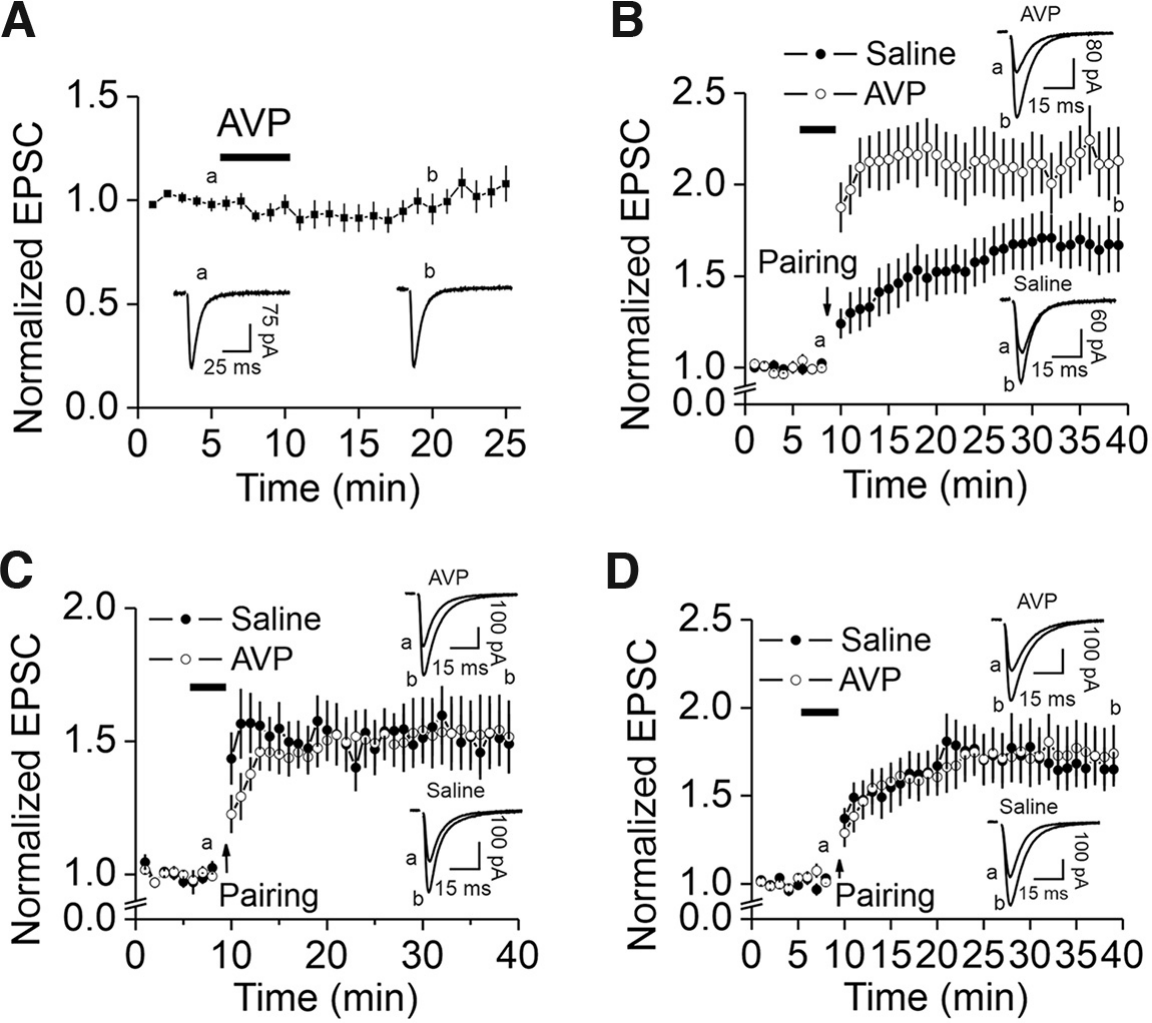
AVP does not modulate basal glutamatergic transmission but enhances LTP at the PP–GC synapses. ***A***, Bath application of AVP (0.3 μm) did not alter significantly AMPA EPSCs recorded at the PP–GC synapses at −65 mV. The stimulation frequency was 0.1 Hz. The extracellular solution contained 10 μm bicuculline, and the intracellular solution was the K^+^-gluconate solution supplemented with 1 mm QX-314. The current traces were the averages of 1 min indicated at the time points shown in the figure. The stimulation artifacts were blanked. ***B***, Bath application of AVP (0.3 μm) significantly enhanced LTP induced by pairing presynaptic stimulation (1 Hz, 40 pulses) with postsynaptic depolarization (−30 mV) recorded with K^+^-gluconate-containing intracellular solution. After recording basal AMPA EPSCs at −65 mV with the stimulation frequency of 0.1 Hz for 5 min, the bath was perfused with the extracellular solution containing AVP (0.3 μm) or saline (0.9% NaCl used to dissolve AVP) for 3 min, and the pairing protocol (1 Hz, 40 pulses, postsynaptic depolarization to −30 mV) was applied in the presence of AVP or saline. Recordings of AMPA EPSCs (−65 mV, 0.1 Hz) were resumed in the extracellular solution to observe the expression of LTP. Current traces were the averages in 1 min at the time points indicated in the figure. ***C***, Application of AVP failed to enhance LTP when Cs^+^-gluconate-intracellular solution was used. ***D***, Application of AVP did not augment LTP in the extracellular solution containing glibenclamide (100 μm) when K^+^-gluconate-intracellular solution was used.

The Ca^2+^ concentration in the extracellular solution was 2.5 mm. At this extracellular Ca^2+^ concentration, bath application of AVP has been shown to depress the slope of field EPSPs recorded in the DG (Chen et al., 1993). We therefore recorded AMPA EPSCs from the GCs by placing a stimulation electrode in the molecular layer to stimulate the PP. Bath application of AVP did not significantly modify AMPA EPSCs at the PP–GC synapses (104 ± 7% of control, *n* = 11; *p *=* *0.577, Wilcoxon test; Fig. 6*A*), suggesting that AVP exerts no significant effect on basal glutamatergic transmission at the PP–GC synapses.

### AVP increases LTP at the PP–GC synapses

In pancreatic β-cells, hyperglycemia results in the closure of K_ATP_ channels, leading to membrane depolarization. Membrane depolarization opens Ca^2+^ channels to increase insulin release to decrease blood glucose concentration. Our results indicate that AVP-mediated activation of V_1a_ receptors elicited subthreshold depolarization of the DG GCs via depression of K_ATP_ channels. At the PP–GC synapses, administration of a pairing protocol has been shown to induce LTP (Colino and Malenka, 1993). We therefore tested the effect of AVP on LTP at the PP–GC synapses by using a protocol of pairing presynaptic stimulation (1 Hz, 40 pulses) with postsynaptic depolarization to −30 mV. With K^+^-gluconate-containing intracellular solution, application of the protocol induced LTP (30 min after the protocol, 167 ± 15% of control, *n* = 13; *p *=* *0.0002 vs baseline, Wilcoxon test; Fig. 6*B*). We further explored the effects of AVP on LTP at the PP–GC synapses. After recording basal AMPA EPSCs for 5 min, AVP (0.3 μm) dissolved in the extracellular solution was applied for 3 min because our results showed that the maximal effect of AVP on DG GCs could be observed in this time period. We then applied the protocol in the continuous presence of AVP. Under these circumstances, the level of LTP was significantly increased (213 ± 18% of control, *n* = 14; *p *=* *0.0001 vs baseline, Wilcoxon test; *F*_(1,950)_ = 194.8; *p *<* *0.0001 vs control LTP, two-way ANOVA; Fig. 6*B*), suggesting that AVP augments LTP.

Because our results indicate that the activation of V_1a_ receptors depolarizes the DG GCs via depression of K_ATP_ channels, we then tested the roles of K^+^ channels by using Cs^+^-gluconate-containing intracellular solution to annul the contribution of K^+^ channels. In this condition, the application of AVP did not significantly increase LTP (152 ± 14% of control, *n* = 11; *p *=* *0.001 vs baseline, Wilcoxon test; Fig. 6*C*), compared with saline (151 ± 15% of control, *n* = 10; *p *=* *0.002 vs baseline, Wilcoxon test; *F*_(1,722)_ = 0.106; *p *=* *0.745 vs LTP in response to AVP, two-way ANOVA; Fig. 6*C*), suggesting that AVP-mediated depression of K^+^ channels is responsible for AVP-elicited augmentation of LTP. We further probed the roles of K_ATP_ channels in AVP-induced enhancement of LTP with the K^+^-containing intracellular solution. In the presence of the K_ATP_ channel blocker glibenclamide (100 μm), the application of AVP did not significantly alter LTP induced by the administration of the pairing protocol (174 ± 16% of control, *n* = 11; *p *=* *0.001 vs baseline, Wilcoxon test; Fig. 6*D*), compared with the LTP in response to bath application of saline (165 ± 10%, *n* = 11; *p *=* *0.001 vs baseline, Wilcoxon test; *F*_(1,760)_ = 0.039; *p *=* *0.843 vs the LTP in response to AVP, two-way ANOVA; Fig. 6*D*). These results together suggest that AVP-induced depression of K_ATP_ channels contributes to its facilitatory effect on LTP.

## Discussion

Our results indicate that application of AVP induces an inward current recorded from the DG GCs in voltage clamp. In current-clamp mode, AVP depolarizes the DG GCs and increases the action potential firing numbers. The effects of AVP are mediated by activation of V_1a_ receptors and require the function of PLCβ. Whereas intracellular Ca^2+^ release and PKC activity are unnecessary, PLCβ-elicited depletion of PIP_2_ is responsible for AVP-elicited excitation of the DG GCs. AVP-induced excitation of the DG GCs is mediated by the depression of K_ATP_ channels. Activation of V_1a_ receptors augments LTP at the PP–GC synapses, which is also mediated by the depression of K_ATP_ channels. Our results provide a cellular and molecular mechanism to explain the roles of V_1a_ receptor activation in learning and memory and anxiety.

Our results show that AVP-elicited excitation of the DG GCs is mediated by the depression of K_ATP_ channels. Consistent with our electrophysiological results, high densities of K_ATP_ channels are expressed in the DG GCs (Mourre et al., 1990; Zawar et al., 1999; Pelletier et al., 2000; Tanner et al., 2011). K_ATP_ channels play a key role in the coupling between cellular metabolism and electrical activity in a wide range of tissues. K_ATP_ channels are formed from an ATP-binding cassette protein, the sulfonylurea receptor (SUR1, SUR2), and a Kir channel (Kir6.1, Kir6.2). Both subunits assemble in a 1:1 stoichiometry, with four SUR and four Kir subunits required to form functional K_ATP_ channels (Inagaki et al., 1995, 1997; Clement et al., 1997; Shyng and Nichols, 1997). While Kir6 acts as the pore-forming part in the channel complex that determines its single-channel conductance, its blockade by polyamines, and its inhibition by ATP, SUR has been identified as the regulatory subunit of K_ATP_ channels that confers sensitivity to sulfonylureas, channel openers, and Mg-ADP (Baukrowitz and Fakler, 2000). The AVP-sensitive currents in the DG GCs show inward rectification, and application of the K_ATP_ channel blocker glibenclamide induces an inward current by itself and blocks the effects of AVP, suggesting that the activation of V_1a_ receptors excites the DG GCs via inhibition of K_ATP_ channels.

Our results further demonstrate that AVP-elicited excitation of the DG GCs is mediated by the activation of V_1a_ receptors, consistent with the expression of high densities of AVP receptors in the GC (Brinton et al., 1984; De Kloet et al., 1985; van Leeuwen et al., 1987; Campbell et al., 2009). Our results further demonstrate that PLCβ is required, whereas intracellular Ca^2+^ release and PKC are dispensable for AVP-elicited inward currents in the DG GCs. In line with our results, exogenous application of IP_3_ had no effect on K_ATP_ channel activity (Fan and Makielski, 1997; Shyng and Nichols, 1998). However, it is controversial as to whether PKC is involved in modulating K_ATP_ channels. Whereas PKC has been shown to inhibit recombinant (Thorneloe et al., 2002) and native (Bonev and Nelson, 1993; Hatakeyama et al., 1995; Nuttle and Farley, 1997; Jun et al., 2001) K_ATP_ channels, PKC is not required for muscarinic suppression of K_ATP_ channels mediated by the M3/G_q/11_/PLC pathway in mouse ileal smooth muscle cells (Wang et al., 2018).

PIP_2_ has been shown to modulate numerous ion channels (Suh and Hille, 2008; Rodríguez-Menchaca et al., 2012), including the K_ATP_ channels (Baukrowitz and Fakler, 2000). PIP_2_ is known to increase the open probability and decrease the ATP sensitivity of the K_ATP_ channels (Hilgemann and Ball, 1996; Fan and Makielski, 1997; Baukrowitz et al., 1998; Shyng and Nichols, 1998). Low-micromolar ATP is sufficient to inhibit K_ATP_ channels following patch excision, whereas millimolar concentrations of the nucleotide are required for channel inhibition after PIP_2_ is applied to inside-out patches for a few seconds and prolonged exposure to PIP_2_ renders the channels completely insensitive to 1 mm ATP (Baukrowitz et al., 1998; Shyng and Nichols, 1998). Membrane PIP_2_ content increases when PI and PI 4-monophosphate (PIP) are consecutively phosphorylated by PI 4-kinase and PIP 5-kinase (Anderson et al., 1999), whereas dephosphorylation of PIP_2_ mediated by inositolpolyphosphate phosphatase decreases PIP_2_ content in the membrane (Majerus et al., 1999). In addition, PIP_2_ is hydrolyzed by PLC to generate IP_3_ and DAG in response to G-protein-coupled receptors or tyrosine kinase receptors, resulting in the reduction of membrane PIP_2_ content by ∼85% (Willars et al., 1998). Because PIP_2_ has been shown to augment the open probability and decrease the ATP sensitivity of the K_ATP_ channels (Hilgemann and Ball, 1996; Fan and Makielski, 1997; Baukrowitz et al., 1998; Shyng and Nichols, 1998), PLCβ-elicited depletion of PIP_2_ in response to V_1a_ receptor activation likely decreases open probability and increases the ATP sensitivity of the K_ATP_ channels. The outcome would be the depression of K_ATP_ channels and excitation of the DG GCs.

In CA1 pyramidal neurons of the hippocampus, the activation of V_1a_ receptors increases neuronal excitability by the inhibition of GIRK channels (Hu et al., 2022), whereas the results in this study indicate that the activation of V_1a_ receptors excites the DG GCs by depressing K_ATP_ channels. The discrepancy may be because of the distinct expression of K_ATP_ channels between CA1 pyramidal neurons and the DG GCs. K_ATP_ channels are expressed in 89% of the GCs, whereas only 26% of CA1 pyramidal neurons express K_ATP_ channels (Zawar et al., 1999).

In the DG, bath application of AVP increased the slope of field potentials when the extracellular Ca^2+^ concentration was 1.5 mm, but decreased it when the extracellular concentration was 2.5 mm (Chen et al., 1993). With whole-cell recordings, we failed to observe significant alteration of AMPA EPSCs in response to bath application of AVP in our extracellular solution containing 2.5 mm Ca^2+^. One explanation for the discrepancy of the results is that field potentials may represent the combined effects of AVP from many synapses, whereas AMPA EPSCs recorded by whole-cell recordings reflect the action of AVP at the synapses onto a single GC. If the effects of AVP on synaptic transmission are subtle, they may have been missed with whole-cell recordings from single cells. However, we have indeed observed that the bath application of AVP significantly increases the level of LTP at the PP–GC synapses by depressing K_ATP_ channels. Because the induction of LTP at the PP–GC synapses is dependent on NMDA receptors (Colino and Malenka, 1993) and NMDA receptors are voltage-dependently blocked by Mg^2+^, AVP-induced depolarization could facilitate NMDA receptor opening and thus augments LTP. An alternative mechanism is that V_1a_ receptor-mediated depression of K_ATP_ channels could depolarize the DG GCs to open voltage-gated Ca^2+^ channels, resulting in the augmentation of Ca^2+^ influx to facilitate LTP. Further studies are required to determine the cellular and molecular mechanisms underpinning AVP-mediated augmentation of LTP. Consistent with our results, the depression of K_ATP_ channels enhances hippocampal LTP (Schröder et al., 2004; Moriguchi et al., 2018, 2021).

The physiological functions underlying V_1a_ receptor-elicited excitation of the DG GCs and facilitation of LTP may be related to the effects of AVP on learning and memory (Alescio-Lautier and Soumireu-Mourat, 1998). For example, microinjection of AVP into the DG facilitates (Kovács et al., 1979), whereas microinjection of AVP antiserum into the dorsal DG attenuates (Kovács et al., 1982), passive avoidance behavior in rats. Intracerebroventricular injection of vasopressin-(4–9), a major metabolite C-terminal fragment of AVP, ameliorates scopolamine-induced impairments of rat spatial memory (Mishima et al., 2001). Subcutaneous injection of NC-1900, an active fragment analog of AVP, improves learning and memory deficits induced by β-amyloid protein in rats (Tanaka et al., 1998). However, the cellular and molecular mechanisms underlying AVP-mediated augmentation of learning and memory have not been determined. Our results that activation of V_1a_ receptors excites the DG GCs and augments LTP at the PP–GC synapses could serve as a cellular mechanism to explain the effects of AVP on memory. Furthermore, activation of V_1a_ receptors exerts anxiogenic effects (Landgraf et al., 1995; Bielsky et al., 2004, 2005; Egashira et al., 2007), and the ventral hippocampus is closely involved in anxiety-like behaviors (Charney and Deutch, 1996; Kjelstrup et al., 2002; Bannerman et al., 2003; Engin and Treit, 2007; Fanselow and Dong, 2010; Adhikari, 2014; Strange et al., 2014; Calhoon and Tye, 2015; Jimenez et al., 2018). Because the DG GCs are glutamatergic neurons and elevation of glutamatergic functions underlies the generation of anxiety and reduction of glutamatergic functions represents a novel treatment for anxiety (Kent et al., 2002; Gorman, 2003; Bergink et al., 2004; Simon and Gorman, 2006; Sanacora et al., 2008; Riaza Bermudo-Soriano et al., 2012; Sanacora et al., 2012), our results may represent a cellular and molecular mechanism whereby the activation of V_1a_ receptors facilitates anxiety responses.

## Synthesis

Reviewing Editor: Lindsay De Biase, University of California Los Angeles Decisions are customarily a result of the Reviewing Editor and the peer reviewers coming together and discussing their recommendations until a consensus is reached. When revisions are invited, a fact-based synthesis statement explaining their decision and outlining what is needed to prepare a revision will be listed below. The following reviewer(s) agreed to reveal their identity: Hau-Jie Yau.

In the article “PLCβ-mediated depletion of PIP2 and ATP-sensitive K+ channels are involved in arginine vasopressin-induced facilitation of neuronal excitability and LTP in the dentate gyrus” the authors investigate the ability of arginine vasopressin (AVP) to modulate intrinsic activity and synaptic transmission of dentate gyrus (DG) granule cells, the mechanisms of which remain elusive. They combine pharmacological tools and bath-application of AVP to acute hippocampal brain slices, and find that this manipulation increases excitability of DG granule cells. Their finding is supported by AVP-induced inward currents measured with whole-cell voltage-clamp recordings from granule cells, and by AVP-induced depolarization and facilitation of action potential firing, determined in current-clamp recordings from granule cells. They found AVP-induced no changes to glutamatergic synaptic signaling onto DG granule cells. These results advance knowledge based on previous studies of AVP effects on hippocampal DG electrical signaling using extracellular field recordings. The authors investigate the underlying mechanism by determining the pharmacological profile of the AVP-induced inward current. They found that PLC activation is required but not IP3-dependent ER Ca2+ release or PKC activation. Instead, they can inhibit AVP effects by inhibiting KATP channels or by providing an excess of the PIP2 analog diC8-PIP2. From these experiments, and the reported high expression of V1A in DG granule cells, they conclude that the AVP effect on granule cell excitability may be explained by V1A-mediated depletion of PIP2, which might render tonically active KATP channels more sensitive to inhibition by ATP and thereby reduce the resting potassium conductance. Finally, they demonstrate that AVP facilitated the expression of PP-GC LTP in a KATP-dependent manner.

The manuscript is well written and organized. With the limitation that the authors did not employ any genetic manipulation of V1a in DG GCs, the experiments appear technically well-designed and the proposed model appears plausible based on prior literature and the reported experiments. The novelty is limited and more incremental due to similar AVP-induced effects reported for CA1 pyramidal neurons, albeit by targeting a different potassium channel (GIRK). Nevertheless, by linking KATP channel function to AVP effects on the gatekeeper of hippocampal function this study advances our insight into the underlying molecular and cellular mechanisms. These findings could also help shed light on molecular mechanisms for AVP modulation of DG-relevant behavioral functions. However, reviewers agree that several concerns in the data need to be addressed.

Major concerns:

1) For Figs. 1, 3, 4 and 5 the authors used the same AVP control effect as reference for statistical comparison (10 direct comparisons). Ideally, each experiment should have its own control group. This reveals critical information about how repeatable and consistent the data are in the control condition if experiments are executed at different times as the project evolves and progresses. Can the authors repeat at least one key experiment (e.g. for Fig. 5) and include a new control condition? At a minimum, it is recommended that in all figures following Fig. 1, the AVP control data be presented as grayed out and the authors explicitly comment on the recycling of the control data in the respective population data description in the figure legends. It should also be clarified whether the recycling has been accounted for in multiple comparison compensations of the statistical analyses.

2) The authors should be more rigorous in choosing their representative trace examples for panels Fig. 1B, Fig. 3A, Fig. 4A and Fig. 5E. The representative examples should be close to the mean value for that entire group.

3) The control experiments in Fig. 5 convincingly demonstrate that ER Ca2+ release and/or PKC activation are not required for the AVP effect studied here. It has been found that 2-arachidonoylglycerol (2-AG) and arachidonic acid, two diacylglycerol (DAG) lipase-dependent downstream products of DAG, can right-shift the voltage-dependence of voltage-gated potassium channels underlying the A-type current (PMID:28262417). It is recommended to include inhibition of DAG lipase as control experiment in Fig. 5.

4) The authors have provided the evidence that there was tonic activation of KATP channels in DG GCs in experimental condition. Therefore, it is unexpected that the manuscript did not provide the key direct evidence that AVP would increase membrane resistance by closing active KATP channels.

Minor concerns:

1) In the presentation and discussion of the LTP results it appears that the authors did not take sufficient efforts to distinguish between induction and expression. Fig. 7 legend emphasizes LTP expression. Specifically, on page 9 lower half they state: “We further explored the effects of AVP on the induction of LTP at the PP-GC synapses. [...], suggesting that AVP augments the expression of LTP.” If the investigators claim that they tested the effect of AVP on induction of LTP they would clearly need to explain how this was possible with the GC membrane potential clamped to -30 mV during induction. Since the LTP experiment was conducted in voltage clamp mode, how can AVP-elicited depolarization ‘facilitate the opening of Ca2+ channels or relieve the Mg2+ blockade of NMDA receptors’? They need to come up with some other reasonable explanations or they could test the AVP modulation of NMDAR-mediated EPSC.

2) page 12, middle: “reflex” probably means “reflects”

3) It is suggested to combine Figure 3 and 4.
